# TSC but not PTEN loss in starving cones of retinitis pigmentosa mice leads to an autophagy defect and mTORC1 dissociation from the lysosome

**DOI:** 10.1038/cddis.2016.182

**Published:** 2016-06-30

**Authors:** A Venkatesh, S Ma, C Punzo

**Affiliations:** 1 Department of Ophthalmology and Gene Therapy Center, University of Massachusetts Medical School, Worcester, MA 01605, USA; 2 Department of Neurobiology, University of Massachusetts Medical School, 386 Plantation Street, Worcester, MA 01605, USA

## Abstract

Understanding the mechanisms that contribute to secondary cone photoreceptor loss in retinitis pigmentosa (RP) is critical to devise strategies to prolong vision in this neurodegenerative disease. We previously showed that constitutive activation of the mammalian target of rapamycin complex 1 (mTORC1), by loss of its negative regulator the tuberous sclerosis complex protein 1 (*Tsc1*; also known as Hamartin), was sufficient to promote robust survival of nutrient-stressed cones in two mouse models of RP by improving glucose uptake and utilization. However, while cone protection remained initially stable for several weeks, eventually cone loss resumed. Here we show that loss of *Tsc1* in the cones of RP mice causes a defect in autophagy, leading to the accumulation of ubiquitinated aggregates. We demonstrate that this defect was not due to an inhibition of autophagy initiation, but due to an accumulation of autolysosomes, suggesting a defect in the end-stage of the process causing an amino-acid shortage in cones, thereby hampering long-term cone survival. Because cells with TSC loss fail to completely inhibit mTORC1 and properly activate autophagy in the absence of amino acids, we sporadically administered the mTORC1 inhibitor rapamycin, which was sufficient to correct the defects seen in cones, further enhancing the efficiency of cone survival mediated by *Tsc1* loss. Concordantly, activation of mTORC1 by loss of the phosphatase and tensin homolog (*Pten*) did not affect autophagy and amino-acid metabolism, leading to a more sustained long-term protection of cones. As loss of *Pten*, which in cones results in less robust mTORC1 activation when compared with loss of *Tsc1*, still affords long-term cone survival, therapeutic interventions with mTORC1 activators or gene therapy with selected mTORC1 targets that improve glucose metabolism are potential strategies to delay vision loss in patients with RP.

Retinitis pigmentosa (RP) is an untreatable inherited retinal degenerative disease that leads to blindness affecting 1:3500 people in the United States. Most individuals with RP have mutations in genes that are exclusively expressed in the night active rod photoreceptors; however, once rod death has progressed beyond a certain critical threshold,^1^ secondary cone death follows leading to loss of daylight, color and high-acuity vision.^2^

We previously proposed that secondary cone degeneration is the result of inadequate nutrient supply caused by structural changes initiated by the loss of the overabundant rods.^1^ Consequently, improving cell metabolism by activation of mammalian target of rapamycin complex 1 (mTORC1), a key kinase that regulates cell metabolism by balancing demand with supply, promoted cone survival, by increased expression of genes involved in glucose uptake, utilization, and retention enabling cones to counteract the conditions of nutrient stress.^1, 3, 4^ Consistent with that loss of mTORC1 activity during disease accelerated cone death as cones failed to balance demand with supply.^3^ Cone survival was also dependent on the persistence and strength of mTORC1 activation. Daily systemic injections of insulin, which desensitize the insulin receptor over time and reduce mTORC1 activity due to the feedback loop within the pathway,^4^ did only promote cone survival for a period of 4 weeks.^1^ In contrast, loss of either of the two negative regulators of mTORC1, *Pten* (phosphatase and tensin homolog) and *Tsc1* (tuberous sclerosis complex protein 1), which circumvent the negative feedback loop, promoted cone survival for a period of up to 8 months.^3^ Because loss of the direct upstream negative regulator of mTORC1, *Tsc1*, led to a more robust activation of mTORC1 in cones than loss of *Pten*,^5^ many retinae with loss of *Tsc1* displayed almost a wild-type distribution of cones at 2 months of age. Remarkably, cone death was brought to a halt for at least 1 month^3^ in the fast retinal degeneration-1 (*rd1*) mouse model of RP, which harbors a mutation in the rod-specific phosphodiesterase-6-*β* gene (*Pde6β*).^6^ As activation of mTORC1 affords a mutation-independent approach to prolong vision,^3^ we investigated why in *rd1* mice with loss of *Tsc1*, cone death resumed between 2 and 4 months of age.

mTORC1 is a critical negative regulator of macroautophagy,^4^ henceforth referred to as autophagy, and a positive regulator of cell growth. Therefore, hypertrophic giant cells and aberrant lysosomes are hallmarks of the human TSC disease pathology.^7^ Recently, growth factor-mediated mTORC1 activation by the TSC complex has been shown to require dissociation of the complex from the lysosome, where mTORC1 resides and is activated.^8^ Interestingly, lack in amino acids, which leads to inactivation and loss of lysosomal mTORC1, results in lysosomal localization of the TSC complex to inactivate any residual mTORC1.^9^ Because of that amino-acid-deprived TSC-null cells fail to completely inactivate mTORC1 and properly activate autophagy,^9^ a process important when balancing resources under nutrient stress conditions. During autophagy cytoplasmic cargo is delivered to double-membrane vesicles called autophagosomes, which fuse with the lysosome to form autolysosomes, where content is degraded and amino acids are freed.^10^ The process is mediated by a wide range of proteins, some of which are constantly turned over, hence their accumulation is a sign of an impaired process.^11^ To study if loss of *Tsc1* in *rd1* cones, while promoting cone survival through strong activation of mTORC1, may have simultaneously introduced an unwarranted secondary problem by preventing autophagy to occur properly,^9^ we analyzed the process of autophagy at 2 months of age, a time point just before when cone death resumes.

## Results

### Impaired autophagy upon loss of *Tsc1* in wild-type mice leads to a progressive decline in cone function and cone-specific proteins

**Figure 1:**
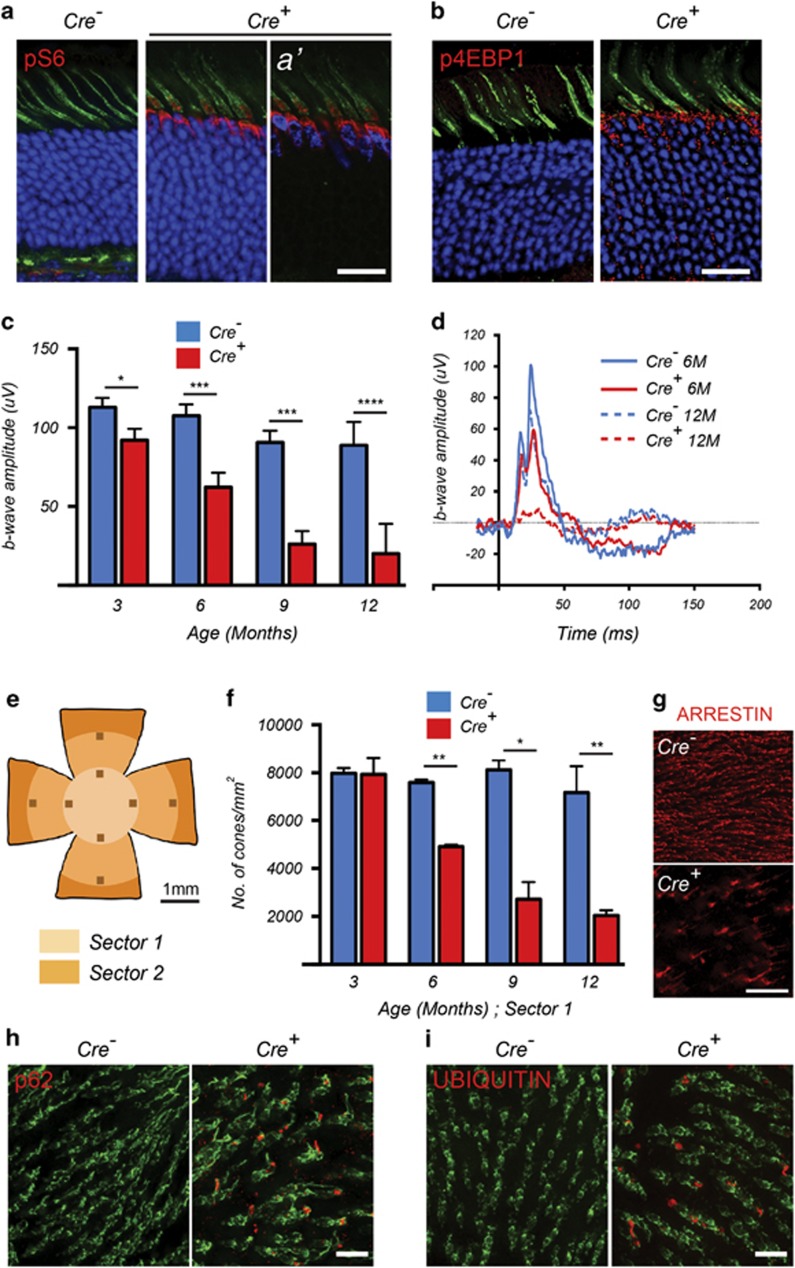
Loss of *Tsc1* in cones of wild-type mice leads to defective autophagy and progressive decline in cone function. Data shown are from mice harboring the *Tsc1^c/c^* allele. (**a** and **b**) Immunofluorescence analyses on retinal cryosections for phosphorylated S6 (**a**) and 4EBP1 (**b**) (red signal) at 2 months of age. Cones were detected by PNA staining (green signal). Blue indicates nuclear DAPI, except in (**a′**), where it indicates expression of the CRE recombinase in cones. (**c**) Evaluation of cone function over time showing average b-wave amplitudes and representative photopic ERG traces (**d**) at indicated time points. Data are representative of recordings from at least six mice per genotype (*P<0.05, ***P<0.001, ****P<0.0001 by Student's *t*-test). (**e–g**) Evaluation of cone survival: schematic of retinal flat mount (**e**) indicating the two sectors (radii: 1 and 2mm) that were used to count cones. (**f**) Bar graphs representing the average number of cone arrestin-positive cones per mm^2^ in Sector 1 over time. Data are representative of at least two mice in each group (*P<0.05, ^**^P<0.01 by Student's *t*-test). (**g**) Representative examples of cone arrestin staining (red signal) from a region in Sector 1 in *Cre*^*−*^ and *Cre*^*+*^ mice at 12 months of age. (**h**) Immunofluorescence analysis on retinal flat mounts (red signal) for p62 and UBIQUITIN (**i**) at 2 months of age. Cones are marked in green by PNA. Scale bars: 20 *μ*m

To study the long-term effect of *Tsc1* loss in cones of mice with retinal degeneration, we first analyzed the effect of its loss in wild-type mice. Mice carrying a conditional knockout allele for *Tsc1* were crossed to the same cone-specific *Cre*-driver line^12^ used in our previous studies (*Tsc1*^*c/c*^_M-opsin-*Cre*, henceforth referred as *Tsc1*^*c/c*^*Cre*^*–*^ or *Tsc1*^*c/c*^*Cre*^*+*^; in all cases *Cre*^*+*^ denotes deletion in cones of the gene indicated). *Tsc1* loss was verified by increased phosphorylation of the mTORC1 targets ribosomal protein-S6 (p-S6) and the eukaryotic translational initiation factor 4E-binding protein (p-4EBP1)^4^ (Figures 1a and b). Electroretinogram (ERG) recordings on mice with *Tsc1* loss showed a strong reduction in cone function over a period of 1 year with a statistically significant decline as early as 3 months of age (Figures 1c and d). We previously showed that the *Cre*-driver line does not affect cone function up to 1 year of age, indicating that the decline in cone function was due to loss of *Tsc1* rather than expression of CRE.^13^ Furthermore, rod function at 1 year of age was not affected upon deletion of *Tsc1* in cones (Supplementary Figure 1a). In agreement with the decline in cone function, a cone count, using an antibody directed against the cone-specific protein cone arrestin, showed significant loss of cone arrestin-positive cones over time (Figures 1e–g and Supplementary Figure 1b).

Impaired autophagy due to constitutive activation of mTORC1 has been shown to affect cellular function and survival in various tissues.^14, 15^ To examine the status of autophagy, we evaluated the expression of p62 (also known as sequestosome-1), a receptor protein involved in the recognition and targeting of autophagic cargo, such as ubiquitinated proteins, to the lysosome for degradation.^16, 17^ We found accumulation of both p62 and ubiquitin in cones of *Tsc1*^*c/c*^*Cre*^*+*^ mice, suggesting a defect in the clearance of ubiquitinated proteins (Figures 1h and i). Western blot analyses with retinal protein extracts did not confirm these findings (Supplementary Figure 1c), likely because cones account only for 3% of all retinal cells, complicating the detection of changes in the levels of ubiquitously expressed proteins or proteins that are preferentially expressed in other retinal cells (Supplementary Figure 1d).^18, 19^

### Loss of *Tsc1* in cones of RP mice causes an accumulation of autolysosomes

**Figure 2:**
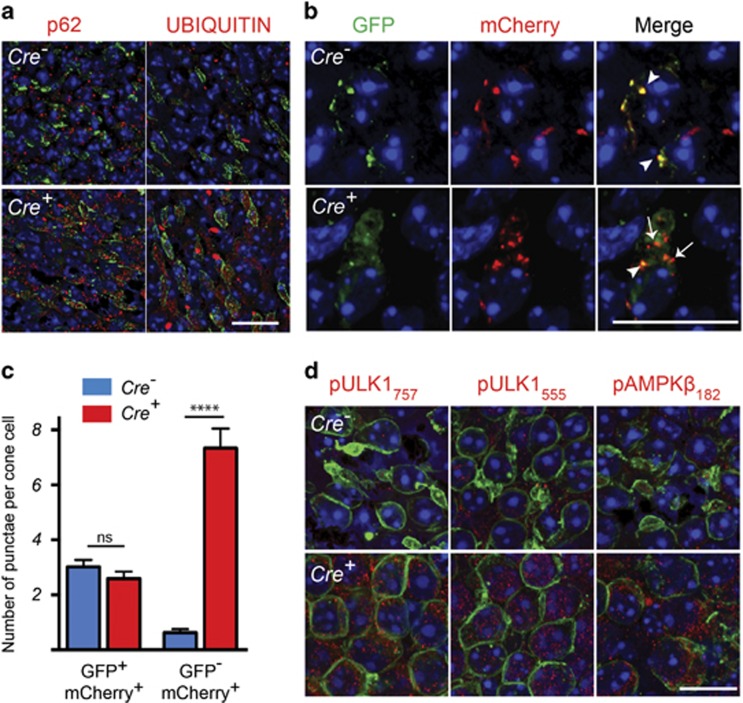
*Tsc1* loss causes an accumulation of autolysosomes. Data shown are from *rd1*-mutant mice harboring the *Tsc1*^*c/c*^ allele at 2 months of age. (**a**) Immunofluorescence analyses on retinal flat mounts for proteins indicated (red signal). Cone layer was identified by PNA staining (green). (**b** and **c**) Immunofluorescence analyses on retinal flat mounts of retinae infected with the AAV9-mCherry-GFP-LC3 vector at birth. (**b**) Representative images at 2 months of age showing increased mCherry^+^/GFP^–^ punctae (arrows) in cones of *Cre*^*+*^ mice indicating an increase in number of autolysosomes. Arrowheads indicate GFP^+^/mCherry^+^ autophagosomes. (**c**) Bar graphs representing average number GFP^+^/mCherry^+^ punctae (autophagosomes) and GFP^-^/mCherry^+^ punctae (autolysosomes) per cone cell. Data are representative of measurements in at least 60 cones over three different animals per genotype (****P<0.0001 by Student's *t*-test). (**d**) Immunofluorescence analyses (red signal) on retinal flat mounts for phosphorylation sites on indicated proteins. Cones were detected by SW OPSIN (green signal) staining. In all panels blue is nuclear DAPI. Scale bars: 20 *μ*m

The findings in wild-type mice led us to assesses if loss of *Tsc1* also impairs autophagy in RP mice (*rd1*-*Tsc1*^*c/c*^*Cre*^*+*^) where cones are subject to conditions of nutrient deprivation.^1, 3^ Similar to our observations in wild-type mice, *rd1-Tsc1*^*c/c*^*Cre*^*+*^ mice showed an accumulation of p62 and ubiquitin aggregates in cones (Figure 2a). To test if autophagy initiation was inhibited, we injected *rd1-Tsc1*^*c/c*^ *Cre*^*−*^ and *Cre*^*+*^ littermates subretinally at birth with a recombinant adeno-associated virus (rAAV9) that expresses a tandem-tagged mCherry-GFP-LC3 gene.^11^ LC3 (microtubule-associated protein-light chain 3) is part of the autophagosomal membrane and remains associated with it even after fusion with the lysosome. Therefore, besides assessing for autophagy initiation, the vector also allows monitoring autophagic flux since GFP is pH sensitive and is quenched by the low pH of the lysosome.^11^ At 2 months of age, we found that while the number of autophagosomes was similar between *Cre*^*−*^ and *Cre*^*+*^ littermates, there was a significant increase in the number of autolysosomes in *rd1-Tsc1*^*c/c*^*Cre*^*+*^ cones (Figures 2b and c). This suggests that loss of *Tsc1* caused a defect in the clearance of autolysosomes, as autophagy was functional up to the stage of autolysosome formation. While the data contradict the notion that activated mTORC1 inhibits autophagy initiation through direct phosphorylation of Ser757 on Unc-51-like autophagy-activating kinase-1 (ULK1), a protein that is part of the autophagy initiation complex,^20, 21^ they are in agreement with a recent report that showed that unlike proliferating cells, postmitotic neurons with TSC loss maintain autophagy through an adenosine monophosphate-activated protein kinase (AMPK)-dependent phosphorylation^22^ of ULK1 at Ser555. Consistent with that we observe increased phosphorylation in *rd1-Tsc1*^*c/c*^*Cre*^*+*^ cones of both ULK1 sites, Ser757 and Ser555, as well as increased phosphorylation of AMPK (Figure 2d), suggesting that the mTORC1-dependent inhibition of ULK1 was overridden by AMPK-dependent activation of ULK1.

**Figure 3:**
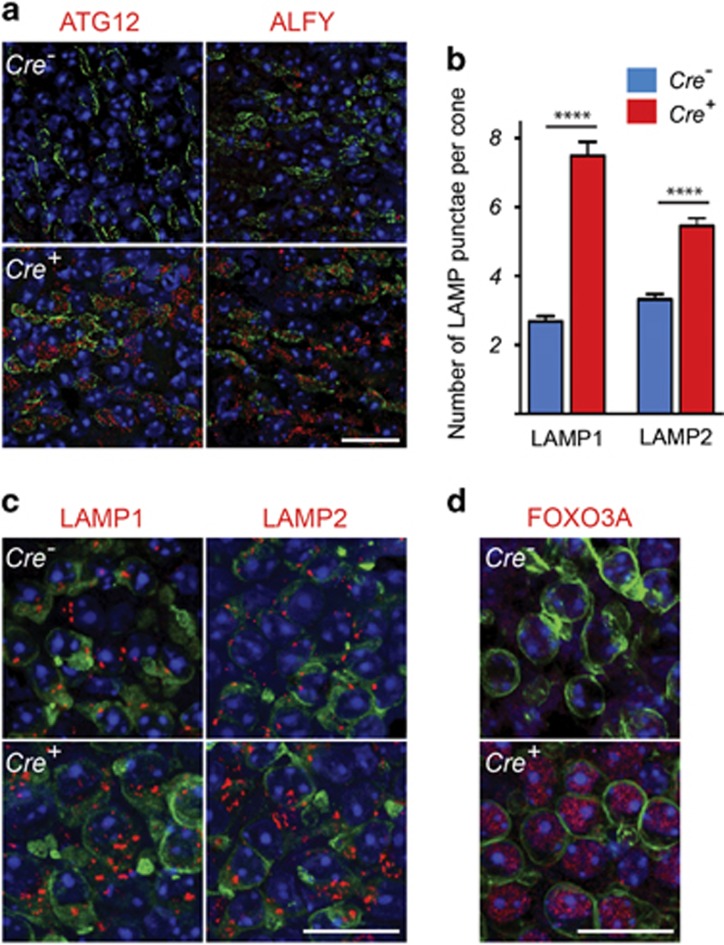
Loss of *Tsc1* in cones of *rd1* mice leads to an increase in autophagy genes and lysosomes. Data shown are from *rd1*-mutant mice harboring the *Tsc1*^*c/c*^ allele at 2 months of age. (**a**) Immunofluorescence analyses on retinal flat mounts for indicated proteins (red signal). Cone layer was identified by PNA staining (green). (**b**) Bar graphs representing average number of LAMP1 and LAMP2 punctae per cone. Values are representative of measurements performed in at least 60 cone cells across two animals per genotype (****P<0.0001 by Student's *t-*test). (**c**) Representative immunofluorescence on retinal flat mounts for LAMP1 and LAMP2 (red signal). Cones were detected by cone ARRESTIN staining (green signal). (**d**) Immunofluorescence analysis of FOXO3A (red signal). Cones were detected by SW OPSIN staining (green signal). In all panels blue signal is nuclear DAPI. Scale bars: 20 *μ*m

In agreement with an increase in the number of autolysosomes, other proteins required for autophagosome formation such as autophagy gene 12 (ATG12), and autophagy-linked FYVE protein (ALFY), a scaffold protein implicated in the selective degradation of ubiquitinated proteins,^23, 24^ were also upregulated in cones (Figure 3a). Quantification of lysosomes by counting the punctae positive for the lysosomal marker proteins lysosomal-associated membrane protein 1 or 2 (LAMP1/2) indicated an increase in the number of lysosomes in *rd1-Tsc1*^*c/c*^*Cre*^*+*^ cones (Figures 3b and c), corroborating the increase in the number of autolysosomes. This increase in the number of lysosomes coincided with increased nuclear expression of the forkhead box protein O3 (FOXO3A), a transcription factor that regulates the transcription of various autophagy-related genes (Figure 3d).^25, 26^ The upregulation of ATG12, ALFY and accumulation of p62 as well as increased expression of FOXO3A was lost upon concurrent removal of *Tsc1* and *Raptor* (regulatory-associated protein of mTOR), suggesting that the increase was mTORC1 dependent (Supplementary Figure 2).

In summary, our data suggest that the defect in autophagy does not arise from autophagy initiation, autophagosome maturation or fusion with the lysosome. As mTORC1 is a negative regulator of the transcription factor EB (*Tfeb*), a master regulator of lysosomal enzymes, the increased accumulation of autolysosomes could result from a deficiency of lysosomal enzymes due to increased mTORC1 activity.^22, 27^

### *Tsc1* loss induces a shortage of free amino acids in cones

**Figure 4:**
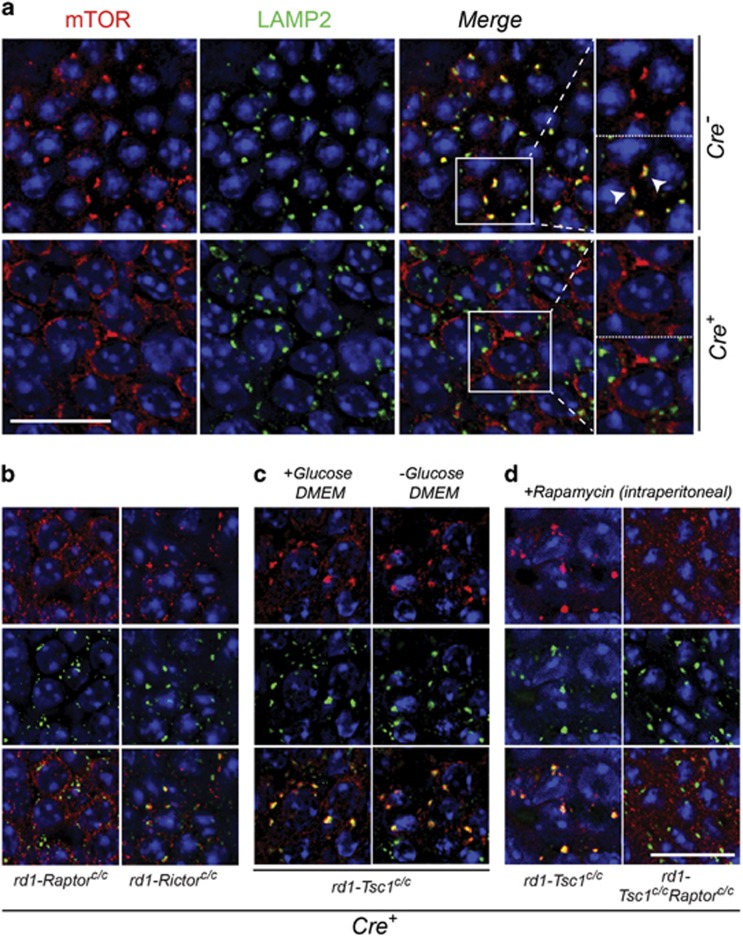
mTOR and LAMP2 colocalization analysis reveals a shortage of amino acids upon *Tsc1* loss in cones. Immunofluorescence analyses on retinal whole mounts of *rd1*-mutant mice harboring the conditional alleles indicated at 2 months of age. (**a**) Localization of mTOR to LAMP2-containing compartments in retinae of *rd1-Tsc1*^*c/c*^ *Cre*^*−*^ mice (arrowheads in higher magnification view). Localization is lost in *Cre*^*+*^ mice (lower row in **a**). Higher magnification view: the upper panel – mTOR; lower panel – mTOR and LAMP2. (**b** and **c**) Only *Cre*^*+*^ retinae of genotypes indicated are shown, with the upper row showing mTOR staining, middle row showing LAMP2 and bottom row showing both mTOR and LAMP2. (**b**) mTOR/LAMP2 colocalization is lost in *rd1-Raptor*^*c/c*^*Cre*^*+*^ mice, but retained in *rd1-Rictor*^*c/c*^*Cre*^*+*^ mice. (**c**) mTOR/LAMP2 colocalization in *rd1*-*Tsc1*^*c/c*^*Cre*^*+*^ mice can be restored by incubating retinae in DMEM media with or without glucose for 2 h. (**d**) Systemic administration of rapamycin (intraperitoneal) also restores mTOR localization at the lysosome, but not in retinae from *rd1*-*Tsc1*^*c/c*^*Cre*^*+*^mice upon concurrent removal of *Raptor.* Retinae were harvested 2 h post-rapamycin injection. In all panels, red staining indicates mTOR, green LAMP2 and blue nuclear DAPI. Scale bars: 20 *μ*m. Higher magnification images in (**a**): × 1.5 original magnification

The autophagy defect in *rd1-Tsc1*^*c/c*^*Cre*^*+*^ cones could cause an imbalance in cellular homeostasis depleting the cell of free amino acids. To test this notion, we exploited the mechanism of amino-acid sensing by mTORC1. Under amino-acid-replete conditions, mTORC1 is recruited by the Ras-related GTPases to LAMP2-containing compartments where it encounters Ras homolog enriched in brain (*Rheb*) for activation.^28^ Therefore, colocalization of mTORC1 with LAMP2 can be used to assess if the cell has a sufficiency of amino acids. We observed at 2 months of age in *Cre*^*−*^ cones distinct focal mTOR staining that was strongly associated with LAMP2. However, in age-matched *rd1-Tsc1*^*c/c*^*Cre*^*+*^ cones mTOR staining was diffuse showing almost no colocalization with LAMP2 (Figure 4a). Because mTOR/LAMP2 colocalization was lost in *rd1-Raptor*^*c/c*^*Cre*^*+*^ retinae, but maintained in *rd1-Rictor*^*c/c*^*Cre*^*+*^ retinae, the colocalization was reflective of mTORC1 staining at the lysosome (Figure 4b).

To test if the absence of mTOR/LAMP2 colocalization in *rd1-Tsc1*^*c/c*^*Cre*^*+*^ cones was caused by a shortage of amino acids, we explanted retinae from *rd1-Tsc1*^*c/c*^*Cre*^*+*^ mice and incubated them in glucose-rich and glucose-free Dulbecco's modified Eagle's media (DMEM). In both cases, short-time exposure to regular media containing amino acids restored the mTOR/LAMP2 colocalization (Figure 4c). Similarly, a single intraperitoneal injection of rapamycin, an allosteric mTORC1 inhibitor, was also able to restore mTOR/LAMP2 colocalization in *rd1-Tsc1*^*c/c*^*Cre*^*+*^ cones, but not in *rd1-Tsc1*^*c/c*^*Raptor*^*c/c*^*Cre*^*+*^ cones (Figure 4d). Taken together, the data suggest that loss of *Tsc1* in cones of RP mice induces an imbalance in the supply and demand of amino acids, which could contribute to the demise of cones see between 2 and 4 months of age.

### Rapamycin reverses the autophagy defect and improves cone survival upon *Tsc1* loss

**Figure 5:**
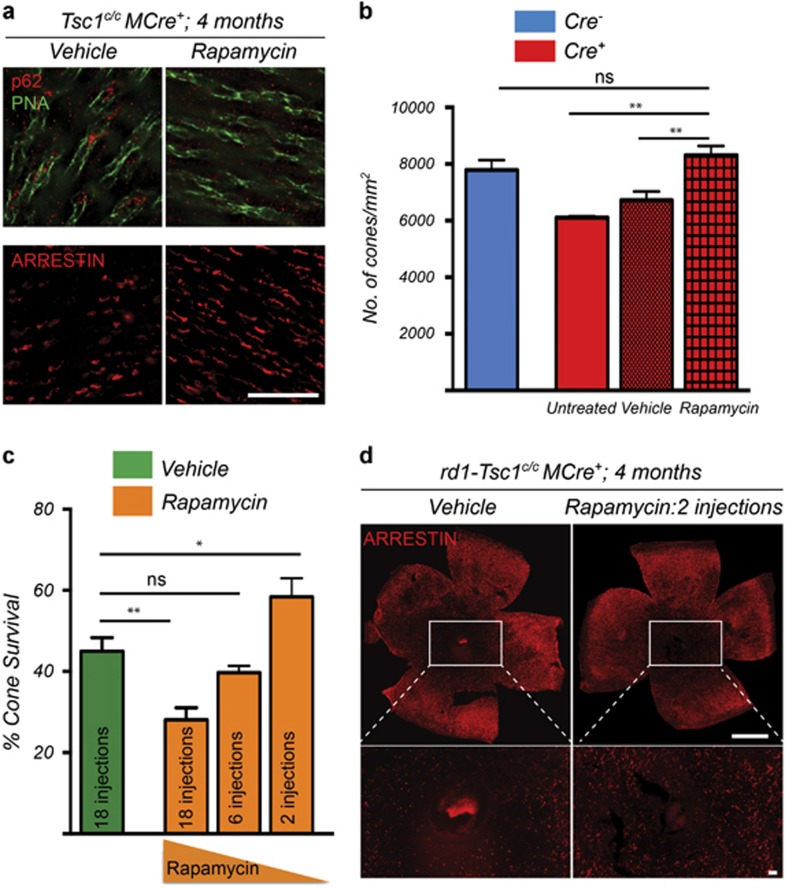
Rapamycin reverses the autophagy defect and improves cone survival upon *Tsc1* loss. (**a**) Immunofluorescence analysis on retinal flat mounts from *Tsc1*^*c/c*^*Cre*^*+*^ mice showing clearance of p62 aggregates in cones (upper panel, red signal; green: PNA) and recovery of cone arrestin (lower panel, red signal) expression upon delivery of rapamycin between P28 to 4 months of age. Images were acquired at 1mm radius from the optic nerve (Sector 1, see Figure 1e). Scale bars: 20 *μ*m. (**b**) Quantification of cone arrestin-positive cones in Sector 1 at 4 months of age of *Tsc1*^*c/c*^*Cre*^*+*^ mice injected with rapamycin or vehicle from P28 onwards (18 injections). Data are representative of at least three mice in each case. **P<0.01 by Student's *t-*test. (**c**) Quantification of cone survival in *rd1*-*Tsc1*^*c/c*^*Cre*^*+*^ mice at 4 months of age upon injection with vehicle or rapamycin starting at P28. Number of rapamycin injections is indicated in bar. Data are representative of at least six mice in each group (*P<0.05, **P<0.01 by Student's *t*-test). (**d**) Representative retinal flat mounts of *rd1*-*Tsc1*^*c/c*^*Cre*^*+*^ mice at 4 months of age showing more central cones when two injections of rapamycin were administered (red signal: cone arrestin; scale bars: 1 mm)

The restoration of colocalization between mTOR and LAMP2 in *rd1-Tsc1*^*c/c*^*Cre*^*+*^ cones upon rapamycin administration (Figure 4c) indicates that autophagy was restored resulting in the release of free amino acids. To test whether the p62 and ubiquitin aggregates or the lack of free amino acids contribute to cone death in *rd1-Tsc1*^*c/c*^*Cre*^*+*^ mice, we performed a long-term rapamycin treatment. Our previous study showed that *rd1-Tsc1*^*c/c*^*Cre*^*+*^ mice display a decline in cone survival between 2 and 4 months of age.^3^ Similarly, removal of *Tsc1* in cones of wild-type mice (*Tsc1*^*c/c*^*Cre*^*+*^) causes loss of cone arrestin expression by 4 months of age. Because loss of mTORC1 activity did not affect cones in a wild-type background but accelerated cone death during disease,^3, 13^ we first administered rapamycin to *Tsc1*^*c/c*^*Cre*^*+*^ mice, where repeated injections between 1 and 4 months of age (18 in total) did clear p62 aggregates and also restored cone arrestin expression (Figures 5a and b).

In *rd1-Tsc1*^*c/c*^*Cre*^*+*^ mice where mTORC1 activity is critical to promote cone survival, we first tested the effect of rapamycin between 1 and 2 moths of age, a time window in which cone survival remains stable at around 78%.^3^ While one injection of rapamycin at 2 months of age causes an increase in free amino acids, it was not able to clear p62 and ubiquitin aggregates (Supplementary Figure 3a). This is because accumulation of p62 and ubiquitin occurs over time because of buildup of autophagic cargo and may require more sustained induction of autophagy for complete clearance. In this regard, repeated injections between 1 and 2 month of age at an interval of 5 days were sufficient to clear p62 and ubiquitin aggregates without affecting cone survival (Supplementary Figures 3a and b). The 5-day interval period was based on the time window it took for p-S6 levels to almost fully recover in cones of *rd1-Tsc1*^*c/c*^*Cre*^*+*^ mice after one rapamycin injection (Supplementary Figures 3c and d). Extended injections of rapamycin (18 in total) up to 4 months of age, however, led to a drop in cone survival when compared with vehicle-injected mice (Figure 5c), suggesting that mTORC1 was inhibited too often over the 3 months time period. We therefore performed two additional treatment regimens reducing the frequency of administration by threefold each time. This led to a dose-dependent increase in cone survival with six injections being at par with vehicle-treated mice and two injections showing a significant improvement in cone survival by 4 months of age (Figures 5c and d). Interestingly, none of the treatment regimens had a negative effect on cone survival in *Cre*^*–*^ mice (Supplementary Figure 3e), suggesting that the amount of mTORC1 activity maintained was sufficient to not alter the natural course of the disease. Postinjection analysis of p62 aggregates at 4 months showed that neither two nor six injections of rapamycin were not sufficient to clear p62 aggregates (Supplementary Figure 4a), suggesting that clearance of p62 is not required to improve cone survival. Consistent with that one injection of rapamycin was sufficient to facilitate autophagy and release free amino acids that directed mTOR to the lysosome for at least 14 days (Supplementary Figure 4b).

### Increased mTORC1 activity by loss of *Pten* is more beneficial for long-term cone survival

**Figure 6:**
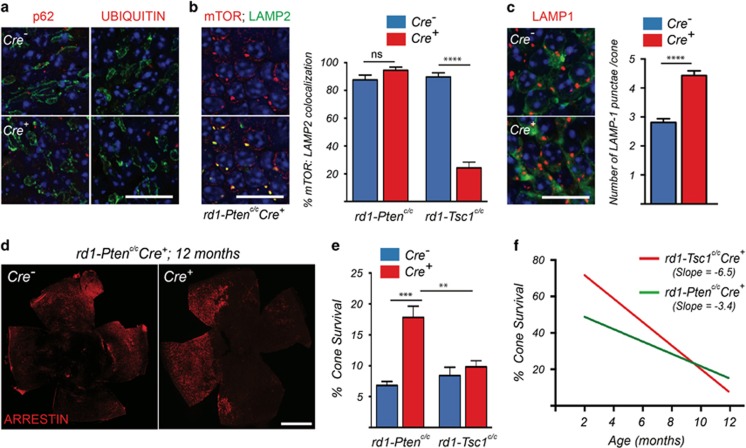
Increased mTORC1 activity by loss of *Pten* is more beneficial for sustained cone survival. Data shown are from *rd1*-*Pten*^*c/c*^ mice, unless indicated otherwise. (**a**) Immunofluorescence analysis on retinal flat mounts for indicated proteins (red signal) at 2 months of age (green: PNA; scale bar: 20*μ*m). (**b**) Immunofluorescence analysis on retinal flat mounts for mTOR (red signal) and LAMP2 (green signal) in *Cre*^*+*^ mice at 2 months of age. Upper panel shows only mTOR and lower panel shows colocalization with LAMP2 (scale bar: 20*μ*m). Bar graphs represent percentage of mTOR punctae that colocalize with LAMP2 per cone. Data from *rd1*-*Tsc1*^*c/c*^*MCre*^*+*^ mice (representative images in Figure 4a) is provided for comparison. The data represent values obtained from at least 60 cones across two animals per genotype (****P<0.0001 by Student's *t*-test). (**c**) Immunofluorescence analysis on retinal flat mounts for LAMP1 (red signal) in *Cre*^*−*^ and *Cre*^*+*^ mice at 2 months of age. Cones were identified by cone arrestin staining (green signal; scale bar: 20*μ*m). Bar graphs showing the number of LAMP1 punctae per cone. Data represent values obtained from at least 60 cones across two animals per genotype (****P<0.0001 by Student's *t*-test). (**d**) Representative retinal flat mounts of *rd1*-*Pten*^*c/c*^ mice at 12 months of age showing improved cone survival in *Cre*^*+*^ mice (red: cone arrestin; scale bar: 1 mm). (**e**) Quantification of cone survival at 12 months of age comparing data obtained from *rd1*-*Pten*^*c/c*^*Cre*^*+*^ mice with previously published data from *rd1*-*Tsc1*^*c/c*^*Cre*^*+*^ mice^3^ (*P<0.05 by Student's *t*-test). (**f**) Linear regression of cone survival over time in *rd1-Tsc1*^*c/c*^*Cre*^*+*^ and *rd1*-*Pten*^*c/c*^*Cre*^*+*^ mice. **P<0.01; ***P<0.005

The TSC complex has recently been shown to be required for full inactivation of mTORC1 under amino-acid-deprived conditions.^9^ Because loss of *Pten* also promoted cone survival^3^ through constitutively activated mTORC1, albeit to a lesser extent than loss of *Tsc1*^3^ due to less activation of mTORC1 in cones,^5^ we investigated the effect of *Pten* loss on autophagy, mTORC1 localization and long-term cone survival. Interestingly, in *rd1-Pten*^*c/c*^*Cre*^*+*^ retinae we found no accumulation of p62 or ubiquitin in cones at 2 months of age (Figure 6a). Concordantly, mTOR/LAMP2 colocalization in cones was similar between *Cre*^*−*^ and *Cre*^*+*^ littermates (Figure 6b), with a less strong increase in lysosomes per cone when compared with loss of *Tsc1*, as assessed by LAMP1-positive punctae (Figure 6c). In agreement with these findings, we did not observe an apparent increase in ULK1 phosphorylation at both sites nor in AMPK phosphorylation (Supplementary Figure 5a) when compared with loss of *Tsc1*, suggesting that autophagy is maintained at a steady state.

In wild-type mice, we observed only occasional accumulation of p62 in cones of *Pten*^*c/c*^*Cre*^*+*^ mice when compared with mice where *Tsc1* was removed in cones (Supplementary Figure 5b). This accumulation was not detrimental to cone function and survival up to 1 year of age (Supplementary Figures 5c and d). Finally, evaluation of long-term cone survival at 1 year of age revealed a twofold increase in the number of cones in *rd1-Pten*^*c/c*^*Cre*^*+*^ mice when compared with *Cre*^*–*^ littermates. Cone survival was also significantly higher when compared with loss of *Tsc1* (Figures 6d and e), which did not show any protective effect at 1 year of age in our previous study.^3^ A linear regression analysis comparing the decline of the protective effect between loss of *Tsc1* and loss of *Pten* showed that while the initial effect upon loss of *Pten* is less robust, the decline over time is not as steep when compared with loss of *Tsc1* (Figure 6f). In summary the data indicate that any increase in mTORC1 activity is beneficial for long-term cone survival as long as autophagy is allowed to progress normally.

## Discussion

Here we set out to understand why loss of *Tsc1* in cones of *rd1* mice promotes initially robust cone survival that remains stable for over a month and then leads to a decline in the number of surviving cones. We found that loss of *Tsc1* results in the accumulation of autolysosomes causing a shortage of free amino acids in cones, which could have ultimately contributed to cone death. Similar to findings in other neurons,^13, 22^ cones with loss of TSC use an AMPK-dependent mechanism to maintain autophagy flux, despite mTORC1-mediated inhibition of autophagy initiation. This contrasts the finding in non-neuronal cells where loss of TSC completely inhibits autophagy.^22^ To compensate for the lack of autophagy and keep up with the increased demand for protein degradation due to the increase in protein synthesis, non-neuronal TSC-null cells appear to increase proteasomal activity.^29^ Consequently, non-neuronal TSC-null cells in which the proteasome is inhibited suffer from an intracellular shortage of free amino acids. While we did not analyze the status of the proteasome, cones in *rd1* mice with *Tsc1* loss increase autophagy similar to other neurons likely to compensate for the increase in protein sysnthesis.^22^ Consistent with an increase in autophagy in cones, we found increased nuclear localization of FOXO3A, a transcription factor that regulates the expression of autophagy and lysosomal-related genes.

The accumulation of autolysosomes seen in *rd1-Tsc1*^*c/c*^*Cre*^*+*^ cones indicates a failure to digest the autolysosomal content, which could be caused by a lack of sufficient lysosomal enzymes. However, it remains to be determined if this was due to mTORC1-mediated inhibition of *Tfeb.*^27, 30^ Nonetheless, the accumulation of autolysosomes and p62-positive aggregates may provide an explanation for the observed neuronal dysfunction in TSC. It may also explain why these neurons are more sensitive to stress, as they cannot dilute undigested aggregates through cell division.^7, 31, 32^ In our case, the added stress condition to the loss of TSC in cones was the disease condition itself, which leads to a nutrient shortage in cones. Thus, lack of amino acids in cones of *rd1* mice upon loss of *Tsc1* may be a disease-specific condition not seen in other neurons with TSC loss. As two administrations of rapamycin were sufficient to further improve cone survival without clearing p62 aggregates, our experiments suggest that the lack of amino acids may have been a more critical factor for cone death than the accumulation of p62 aggregates.

Recent studies have shown that under various stress conditions, TSC is required to localize mTORC1 away from the lysosome for complete shut off.^9, 33^ Thus, in proliferating cells lacking TSC, mTORC1 is still localized at the lysosome and activity is maintained even under conditions of amino-acid deprivation.^9^ Consistent with that, we found 20% of mTORC1 colocalized with LAMP2 in *rd1-Tsc1*^*c/c*^*Cre*^*+*^ cones at 2 months of age. The difference in the extent of mTORC1 localization at the lysosome between the studies may be attributed to differences in experimental time windows, *in vivo versus in vitro*, and/or differences in mTOR regulation between proliferating cells and neurons.^22^ While in our study the reduced mTORC1/LAMP2 colocalization indicates a shortage of amino acids, the 20% of active mTORC1 localized at the lysosome is likely causing the autophagy defect. In contrast to *Tsc1*-null cells, *Pten*-null cells are able to turn off the mTORC1 activity,^9^ because of the presence of a functional TSC. This explains why *Pten* loss in cones results in a more sustained long-term survival effect. Consistent with that in our previous study, we found fewer p-S6-positive cones upon loss of *Pten* than upon loss of *Tsc1.*^3^ Thus, *rd1-Pten*^*c/c*^*Cre*^*+*^ cones, where S6 phosphorylation was not detected, may be the ones where the mTORC1 activity was turned off intermittently to induce autophagy.

In summary, our data show that mTORC1-mediated cone survival is directly dependent on the magnitude of mTORC1 activation. Thus, treatments with mTORC1 activators that do not interfere with TSC or gene therapy with mTORC1 target genes should be beneficial for long-term cone survival as even moderate activation of mTORC1 by loss of *Pten* affords long-term protection.

## Materials and Methods

### Animals

All procedures involving animals were in compliance with the Association for Research in Vision and Ophthalmology Statement for the Use of Animals in Ophthalmic and Vision Research and were approved by the Institutional Animal Care and Use Committees of the University of Massachusetts Medical School. Animals were maintained on a 12-h light/12-h dark cycle with unrestricted access to food and water. Lighting conditions were kept constant in all cages, with illumination ranging between 10 and 15 lux. The *Pten*^*c/c*^, *Tsc1*^*c/c*^, *Raptor*^*c/c*^, *Rictor*^*c/c*^ mice and the cone-specific Cre line have all been described previously.^12, 34, 35, 36, 37^ Genotyping was performed as described in the original publications. In all instances, *Cre*^*+*^ and *Cre*^*−*^ littermates were used for analysis. All mice were genotyped for the absence of the *rd8* allele with mutation in the *Crumbs 1* gene^38^ and none of the mice analyzed were albino.

### Rapamycin administration

Rapamycin was diluted in 50% ethanol to 10 mg/ml. Before injection, the solution was diluted to a concentration of 2 mg/ml in 50% ethanol. All mice were treated with 2 mg/kg body weight of rapamycin by intraperitoneal injections. Mice were either injected at 5-day intervals beginning at P28 until 2 or 4 months of age or at P28, P35, P49, P63, P77 and P105 for the six-injection regimen, or at P28 and P90 for the two-injection regimen. Fifty percent ethanol was used as the vehicle control.

### Retinal explant cultures

Retina was dissected free from other ocular tissue in PBS and then incubated either in regular DMEM media or glucose-free DMEM media. Fetal calf serum was added at 10% (vol/vol) in both cases. Incubation was performed for 2 h at 37 °C and 5% CO_2_. Thereafter, retinae were fixed and processed for antibody staining as described.

### Autophagy flux, mTOR and LAMP quantifications

The mCherry-GFP-LC3 vector^39^ (Plasmid: Claudio Hetz; packaging: UMass Vector Core, rAAV9) was injected subretinally at birth (1 *μ*l of 1 × 10^13^ gc/ml) as described previously.^40^ Retinae were harvested at 2 months of age and processed as described. Images from *Cre*^*−*^ and *Cre*^*+*^ retinae were acquired (100 ×) at the same exposure and manual counting of the GFP and mCherry spots was performed in a blind manner. The same method was used to assess mTOR and LAMP2 colocalization as well as for LAMP1/2 counting. All images were acquired on a Leica DM5500 fluorescence microscope (Leica, Wetzlar, Germany).

### Electroretinography

ERG was performed using the Espion E3 console in conjunction with the ColorDome (Diagnosys LLC, Lowell, MA, USA) as described previously.^13^

### Histological methods

Antibody staining on retinal cryosections and retinal flat mounts were performed as described previously,^3, 40^ except that Triton was replaced by 0.1% saponin for staining procedures involving autophagy and lysosomal proteins. All antibody stainings were performed in PBS buffer. The following primary antibodies and concentrations were used: rabbit *α*-p-S6 (Ser240) (1:300; catalog no. 5364); rabbit *α-*p-4EBP1 (1:300; catalog no. 2855); rabbit *α*-FOXO3A (1:300; catalog no. 12829); rabbit *α*-mTOR (1:500; catalog no. 2983); rabbit *α*-p-ULK1 (Ser757) (1:300; catalog no. 6888); rabbit *α*-p-ULK1 (Ser555) (1:300; catalog no. 5869); rabbit *α*-p-AMPK*β*1 (Ser182) (1:300; catalog no. 4186); rabbit *α*-ATG12 (1:300; catalog no. 2011) all from Cell Signaling Technology (Danvers, MA, USA); rat *α*-LAMP1 (1:500; catalog no. 1D4B-c) and rat *α*-LAMP2 (1:500; catalog no. GL2A7-c) from Developmental Studies Hybridoma Bank (Iowa City, Iowa, USA); guinea-pig *α*-p62 (1:300; catalog no. GP62-C) from Progen (Heidelberg, Germany); goat *α*-short wave opsin (SW OPSIN) (1:500; catalog no. 14363) from Santa Cruz (Dallas, TX, USA); rabbit *α*-ALFY (WDFY3) (1:500; catalog 84888) from Abcam (Cambridge, MA, USA); rabbit *α*-cone arrestin (1:500; catalog no. 15282) and mouse *α*-ubiquitin (1:500; catalog no. 1510) from EMD Millipore (Billerica, MA, USA); mouse *α*-CRE (1:500; catalog no. MMS-106P) from Covance (catalogue no. 900901, Biolegend, San Diego, CA, USA); fluorescein-labeled peanut agglutinin lectin (PNA) (1:500; catalog no. FL-1071) from Vector Laboratories (Burlingame, CA, USA). Nuclei were counterstained with 4′, 6-diamidino-2-phenylindole (DAPI) (catalog no. 9542) from Sigma-Aldrich (St. Louis, MO USA). All secondary antibodies (donkey) were purchased from Jackson ImmunoResearch (West Grove, PA, USA) and were purified F(ab)_2_ fragments that displayed minimal crossreactivity with other species.

### Quantification of cone survival

Retinal flat mount images for the cone arrestin expression analyses were acquired by tiling individual images taken at × 16 over the entire retinal surface area with an automated scanning stage. It must be noted that dormant cones that no longer express cone arrestin are not detected by this method and hence cone arrestin only serves as a proxy to estimate the number of healthy cones. Quantification in wild-type mice: retinae were divided into two sectors with radii of 1 and 2 mm, respectively (Figure 1e). Cones were counted manually in four squares per sector, each square measuring 40 000 *μ*m^2^, to determine the average cone density per sector and genotype (cones per mm^2^). Quantification in *rd1*-mutant mice: cone survival was evaluated by our previously described method by calculating the surface area of the retina that is covered by the cone arrestin signal to extrapolate the percentage of cone survival.^1, 3, 5^ Colocalization between the cone arrestin staining and the retinal surface area was calculated using the CoLocalizer Pro software (Colocalization Research Software, Switzerland, Japan).^41^ We have previously validated the cone survival quantification obtained by this method with actual cone counting and found it to be a reliable estimate of cone survival during degeneration.^3^

### Western blot analysis

Western blot analysis was performed as described previously^3^ with the following modifications. For the western blots depicted in Supplementary Figure 1, the membrane was blocked for 1 h at room temperature in 5% fat-free dry milk powder, incubated with primary antibody overnight at 4 °C, washed three times for 20 min each wash at room temperature, incubated with an HRP-coupled secondary antibody (1:10 000; Santa Cruz Biotechnology Inc.) for 2 h, and washed three times for 20 min (each wash at room temperature). The signal was detected with SuperSignal West Dura (Pierce Biotechnology, catalogue no. 35076, Thermo Fisher Scientific, Waltham, MA, USA). All incubations were performed in PBS in the presence of 0.1% Tween-20 and 5% fat-free dry milk powder. The following primary antibodies were used: rabbit *α*-p-S6 (1:1000; catalog no. 5364), rabbit *α*-p-4EBP1 (1:1000; catalog no. 2855) and rabbit *α-*S6 (1:1000; catalog no. 2217) from Cell Signaling Technology; guinea-pig *α*-p62 (1:500; catalog no. GP62-C) from Progen; rabbit *α*-CRE (1:2000; catalog no. 69050-3) from Novagen (EMD Millipore); mouse *α*-*β*-tubulin (1:2,000; catalog no. 8328) from Sigma-Aldrich; and mouse *α*-ubiquitin HRP conjugate (1:10 000; catalog no. 14049) from Cell Signaling Technology. For the western blots depicted in Supplementary Figure 3, instead of fat-free milk powder, Odyssey Blocking Buffer (LI-COR) (catalog no. 927-40000) from LI-COR (Lincoln, NE, USA) was used for blocking and antibody incubations. No detergent was added during blocking and antibody incubations. Primary antibodies used were same as above, while secondary antibodies were infrared dye-conjugated (1:10 000; LI-COR). Membrane was scanned on an Odyssey Infrared Scanner from LI-COR to detect signal.

### Statistics

The Student's *t-*test was used for statistical analyses. *P-*values <0.05 were considered statistically significant. All error bars represent the S.E.M.

## Abbreviations

4EBP1: eukaryotic translational initiation factor 4E-binding protein
ALFY: autophagy-linked FYVE protein
AMPK: adenosine monophosphate-activated protein kinase
ATG12: autophagy gene 12
ERG: electroretinogram
FOXO3A: forkhead box protein O3
LAMP: lysosomal-associated membrane protein
LC3: microtubule-associated protein-light chain 3
mTORC: mammalian target of rapamycin complex
Pde6*β*: phosphodiesterase-6-*β*
PNA: peanut agglutinin lectin
PTEN: phosphatase and tensin homolog
rAAV: recombinant adeno-associated virus
Raptor: regulatory-associated protein of mTOR
Rictor: rapamycin-insensitive companion of mTOR
rd1: retinal degeneration-1
Rheb: Ras homolog enriched in brain
RP: retinitis Pigmentosa
TFEB: transcription factor EB
TSC: tuberous sclerosis complex
ULK1: Unc-51-like autophagy-activating kinase-1
SW OPSIN: short wave opsin

## Supplementary Material

Supplementary Informations
