# Supplementary material for: TSC but not PTEN loss in starving cones of retinitis pigmentosa mice leads to an autophagy defect and mTORC1 dissociation from the lysosome
Source: Cell Death Dis. 2016 Jun 30;7(6):e2279–. doi: 10.1038/cddis.2016.182 (PMC5108335; doi:10.1038/cddis.2016.182)
Supplement: Supplementary Informations [file cddis2016182x1.pdf]

Supplementary Data

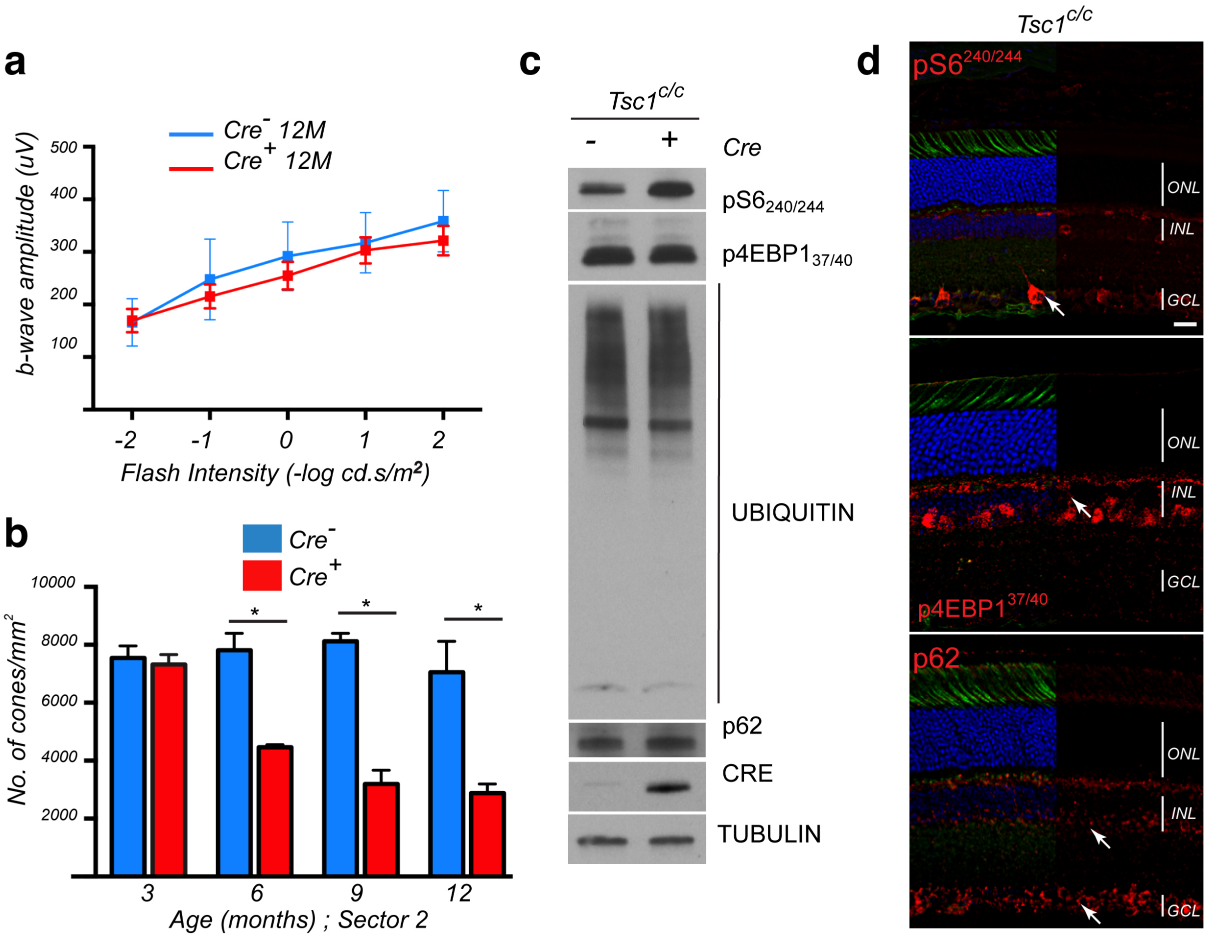

Supplementary Figure 1 Effect of *Tsc1* loss in cones of wild-type mice. Data shown are from wild-type mice harboring the *Tsc1*$^{c/c}$ allele. (**a**) Scotopic ERG recordings at 12 months of age with flashes of increasing intensities showing no significant difference between *Cre*$^{-}$ and *Cre*$^{+}$ mice. Data are representative of recordings from at least 3 mice per genotype. (**b**) Quantification of cone number based on cone arrestin staining at the indicated time points in Sector 2 (see Figure **1e** for schematic of the two sectors; *P < 0.05 by Student's *t* test). (**c**) Western blot analysis of indicated proteins in *Cre*$^{-}$ and *Cre*$^{+}$ mice at 2 months of age showing no appreciable changes between the genotypes apart from increased p-S6 in *Cre*$^{+}$ mice. (**d**) Immunofluorescence analysis on retinal cryosections from *Tsc1*$^{c/c}$ mice at 2 months of age for indicated proteins (red signal; green: PNA; blue: nuclear DAPI). In each panel, 50% of the PNA and DAPI signal is removed to better appreciate the expression of the labeled protein in the different retinal layers (ONL: outer nuclear layer, INL: inner nuclear layer, GCL: ganglion cell layer; arrows point to expression in INL and GCL; Scale bar: 20μm).

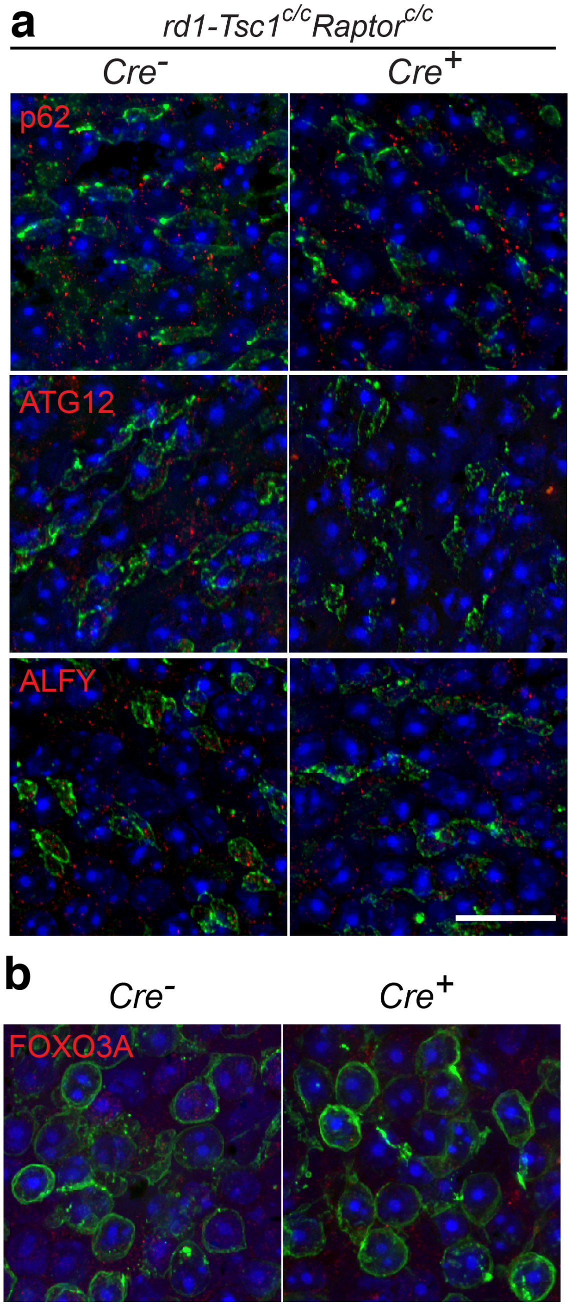

Supplementary Figure 2 Increase in expression of autophagy genes and nuclear FOXO3A is dependent on mTORC1. (**a**) Immunofluorescence analyses on retinal flat mounts for indicated proteins (red signal) in *rd1-Tsc1*$^{c/c}$*Raptor*$^{c/c}$ mice at 2 months of age showing no difference in expression between *Cre*$^{-}$ and *Cre*$^{+}$ mice. (**b**) Same genotype as in (**a**) showing the dependence of nuclear FOXO3A expression (red signal) on mTORC1 activity (green is PNA in (**a**) and SW OPSIN in (**b**); blue: nuclear DAPI; Scale bar: 20μm).

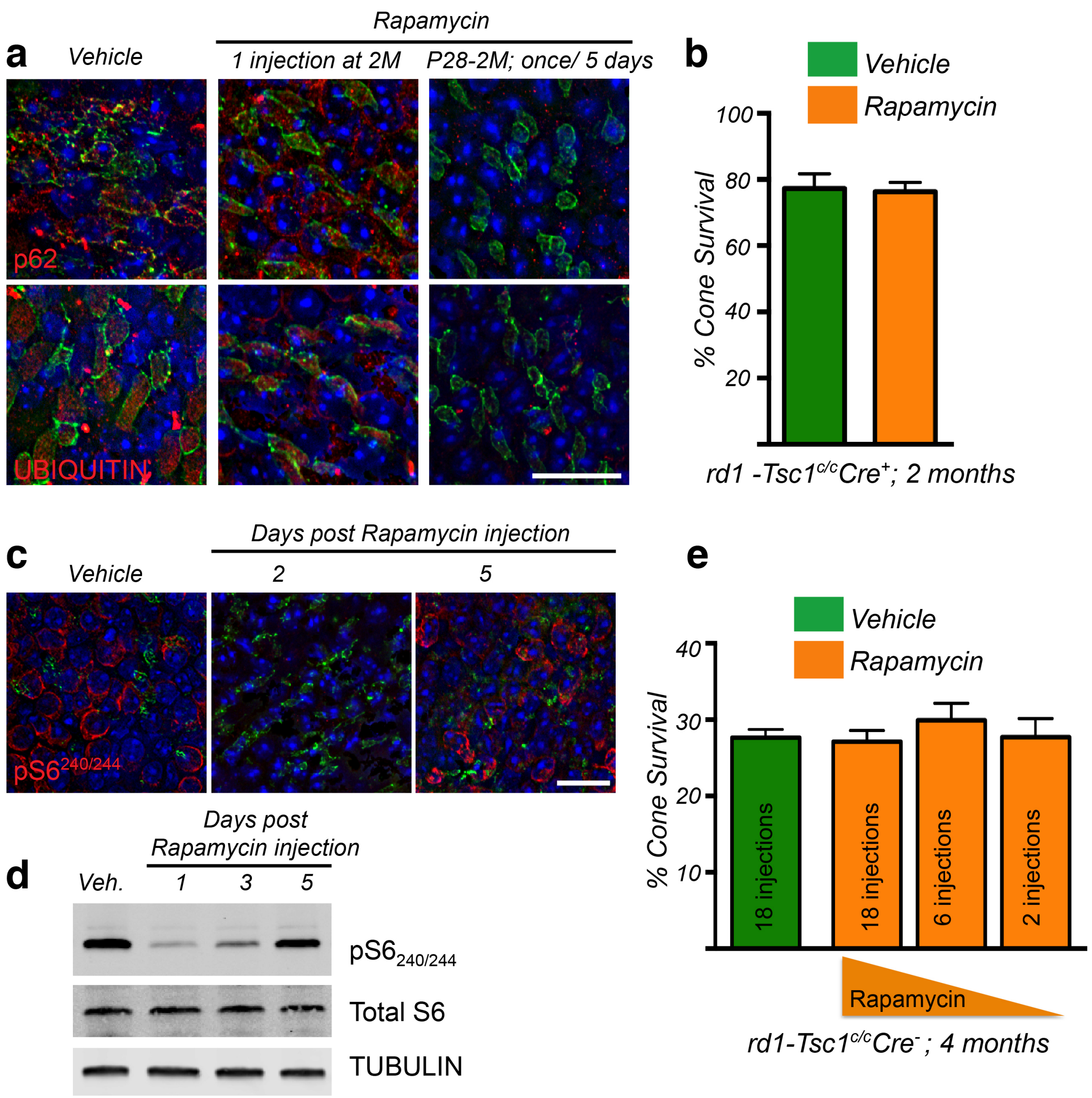

Supplementary Figure 3 Effect of rapamycin on *rd1-Tsc1*$^{c/c}$ mice. (**a**) Immunofluorescence analyses on retinal flat mounts for p62 and UBIQUITIN (red signal) in *rd1-Tsc1*$^{c/c}$*Cre*$^{+}$ mice at 2 months of age upon vehicle or rapamycin administration (Scale bar: 20µm). (**b**) Quantification of cone survival in *rd1-Tsc1*$^{c/c}$*Cre*$^{+}$ mice at 2 months of age when administered with vehicle or rapamycin once every five days from P28 to 2 months. (**c**, **d**) Kinetics of phospho-S6 recovery after one injection of rapamycin in *rd1-Tsc1*$^{c/c}$*Cre*$^{+}$ mice. (**c**) Recovery seen in cones (red signal) by immunofluorescence on retinal flat mounts (green: PNA; Scale bar: 20µm). (**d**) Western blot for phospho-S6 with retinal protein extracts of mice harvested 1, 3 or 5 days after rapamycin injection. By western blot pS6 is almost fully recovered by day 5. (**e**) Quantification of cone survival at 4 months of age in *rd1-Tsc1*$^{c/c}$ *Cre*$^{-}$ mice treated with varying number of rapamycin injections between 1-4 months of age (*Cre*$^{-}$ control of Figure **5c-d**).

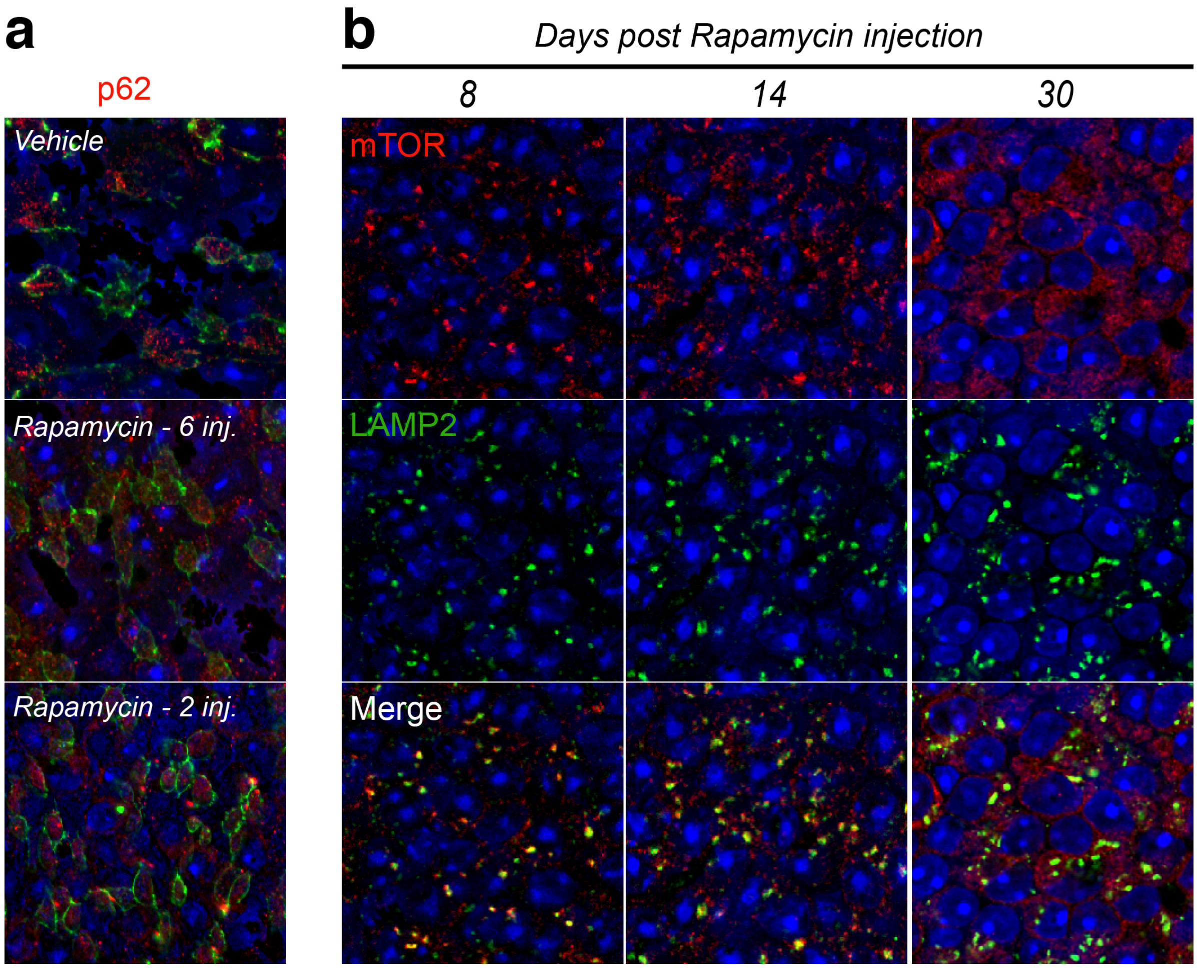

Supplementary Figure 4 Effect of rapamycin administration in *rd1-Tsc1*$^{c/c}$*Cre*$^{+}$ mice. (**a**) Immunofluorescence analyses for p62 (red signal) at 4 months of age in *rd1-Tsc1*$^{c/c}$*Cre*$^{+}$ mice treated with vehicle, 6 and 2 injections of rapamycin (green: PNA). (**b**) Dynamic of mTOR (red signal) and LAMP2 (green signal) colocalization post rapamycin injection in *rd1-Tsc1*$^{c/c}$*Cre*$^{+}$ mice with upper row showing mTOR staining, middle row showing LAMP2 and lower row showing overlay of the two.

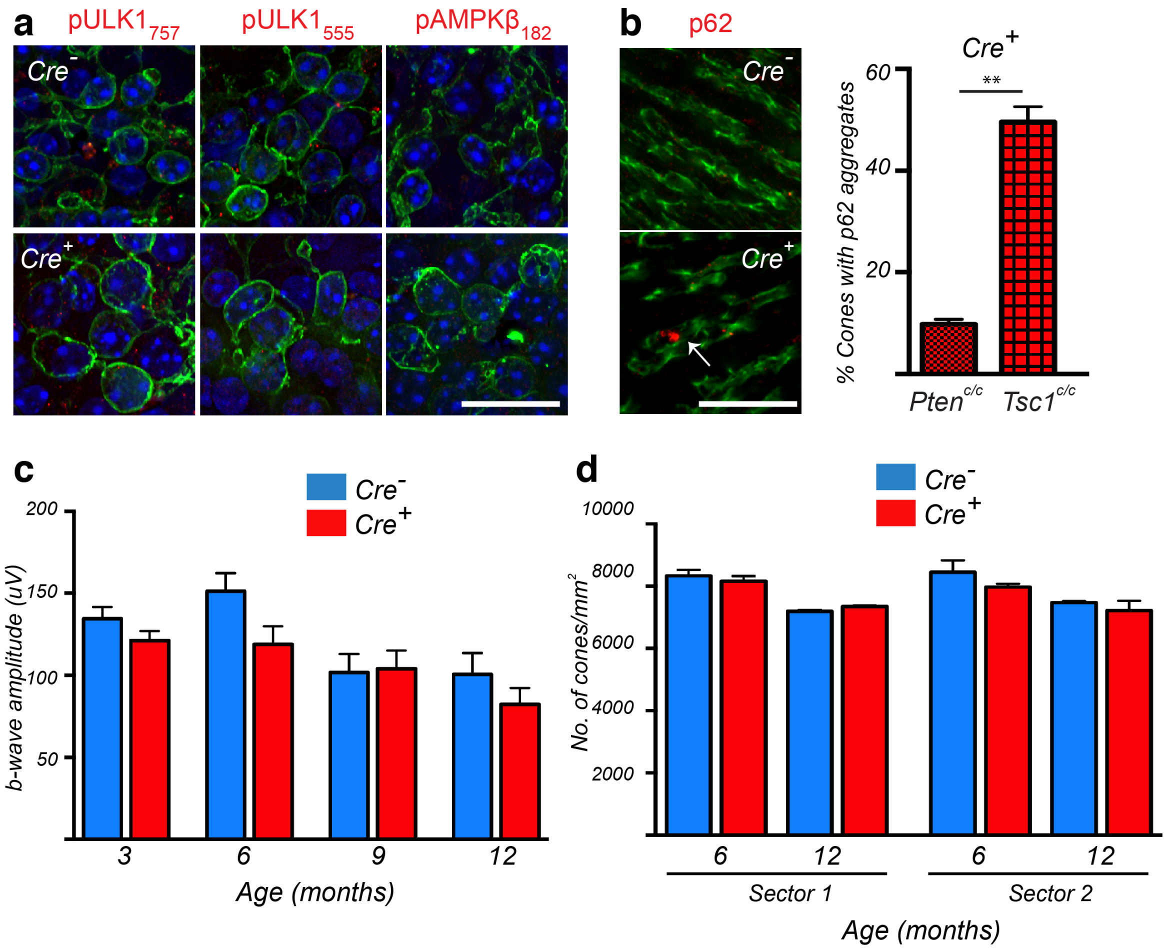

Supplementary Figure 5 Loss of *Pten* in cones of wild-type mice does not affect cone function and expression of cone-arrestin. (**a**) Immunofluorescence analyses (red signal) on retinal flat mounts for phosphorylation sites of indicated proteins in *rd1-Pten*$^{c/c}$ mice. SW OPSIN (green signal) was used to label cones (blue: nuclear DAPI; Scale bar: 20µm). (**b-d**) Analyses in *Pten*$^{c/c}$ mice. (**b**) Immunofluorescence on retinal flat mounts for p62 (red signal) in cones at 2 months of age (arrow: p62 aggregate in cone segment of *Cre*$^{+}$ mouse; green: PNA; Scale bar: 20µm). Bar graph indicating percentage of cones with p62 aggregates in retinae from *Pten*$^{c/c}$*Cre*$^{+}$ and *Tsc1*$^{c/c}$*Cre*$^{+}$ mice at 2 months of age. p62 aggregates were not detected in *Cre*$^{-}$ mice for either genotype and thus not included in the graph. Data are representative of measurements in at least 100 cone segments across 2 animals per genotype (**P < 0.01 by Student's *t* test). (**c**) Evaluation of cone function by photopic ERG recordings from at least 6 mice per group showing b-wave amplitudes over time. No significant difference was detected over time between *Cre*$^{-}$ and *Cre*$^{+}$ littermates. (**d**) Quantification of cone number based on cone arrestin staining in two retinal sectors as described in Figure (**1e**) of at least 2 animals per group. No difference was observed up to 1 year of age between *Cre*$^{-}$ and *Cre*$^{+}$ littermates.
